# Riedel’s lobe of the liver

**DOI:** 10.11604/pamj.2017.28.211.13586

**Published:** 2017-11-07

**Authors:** Ahmed Bensaad, Roberto Algaba

**Affiliations:** 1 CHU Ibn Rochd Casablanca, Morocco; 2 Hopitaux Iris Sud Site Jospeh Bracops Bruxelles, Belgique

**Keywords:** Riedel´s lobe, liver, hepatomegaly

## Image in medicine

We report the case of an 80-year-old woman, presented to our department for an Intestinal obstruction due to postoperative adhesions from an anterior exploratory laparotomy. Preoperative imaging showed a downward elongation of the liver, ending at the level of the iliac crest. Originally reported in 1888 by a German Surgeon named Carl Ludwig Riedel in seven female patients who had palpable masses, they were all confirmed by surgical exploration. The potential complications are rare and include torsion, mass effect morbidity and laparoscopic associated difficulty during different surgical procedures. Unnecessary imaging can be avoided with the knowledge of this normal variant of the right lobe of the liver.

**Figure 1:**
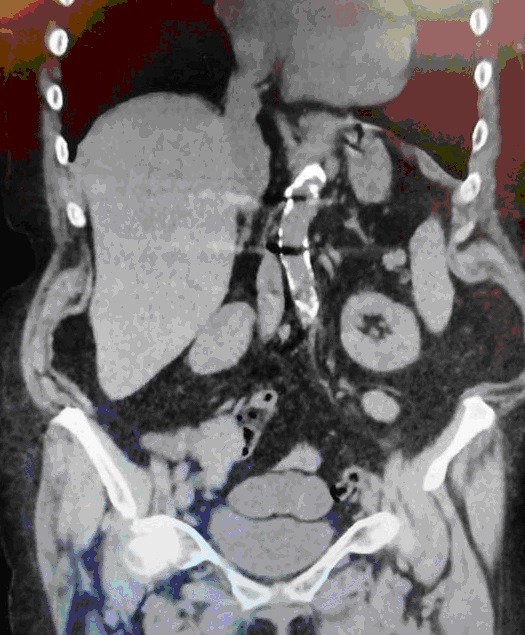
Riedel’s lobe of the liver

